# Basement membrane-associated gene expression as a predictor of survival in oral cancer

**DOI:** 10.1186/s12885-024-12485-2

**Published:** 2024-06-14

**Authors:** Xu Wang, Chaoge Liu, HuiFang Wu, Yulu Gu, Le Zhang, Rongqing Xu, Qing Lin

**Affiliations:** 1 Fujian Provincial Key Laboratory of Brain Aging and Neurodegenerative Diseases, Laboratory of Clinical Applied Anatomy, School of Basic Medical Sciences, Fujian Medical University, Fuzhou, 350122 China; 2 Department of Oramaxillofacial - Head and Neck Surgery, School of Medicine, Tianjin Stomatological Hospital, Nankai University, Tianjin, 300041 China; 3 Tianjin Key Laboratory of Oral and Maxillofacial Function Reconstruction, Tianjin, 300041 China; 4 Department of Oral Pathology, School of Medicine, Tianjin Stomatological Hospital, Nankai University, Tianjin, 300041 China

**Keywords:** Oral cancer, Basement membrane, Risk model, Immune checkpoint

## Abstract

**Background:**

This study sought to investigate the prognostic value of basement membrane (BM)-associated gene expressions in oral cancer.

**Methods:**

We harvested and integrated data on BM-associated genes (BMGs), the oral cancer transcriptome, and clinical information from public repositories. After identifying differentially expressed BMGs, we used Cox and Lasso regression analyses to create a BMG-based risk score for overall survival at various intervals. We then validated this score using the GSE42743 cohort as a validation set. The prognostic potential of the risk scores and their relations to clinical features were assessed. Further, we conducted functional pathway enrichment, immune cell infiltration, and immune checkpoint analyses to elucidate the immunological implications and therapeutic potential of the BMG-based risk score and constituent genes. To confirm the expression levels of the BMG LAMA3 in clinical samples of oral cancer tissue, we performed quantitative real-time PCR (qRT-PCR) and immunohistochemical staining.

**Results:**

The BMGs LAMA3, MMP14, and GPC2 demonstrated notable prognostic significance, facilitating the construction of a BMG-based risk score. A higher risk score derived from BMGs correlated with a poorer survival prognosis for oral cancer patients. Moreover, the risk-associated BMGs exhibited a significant relationship with immune function variability (*P* < 0.05), discrepancies in infiltrating immune cell fractions, and immune checkpoint expressions (*P* < 0.05). The upregulated expression levels of LAMA3 in oral cancer tissues were substantiated through qRT-PCR and immunohistochemical staining.

**Conclusion:**

The BMG-based risk score emerged as a reliable prognostic tool for oral cancer, meriting further research for validation and potential clinical application.

## Background

Oral cancer, predominantly comprising oral squamous cell carcinoma (OSCC), is highly prevalent with high recurrence and mortality rates [1, 2]. OSCC primarily manifests in the upper and lower gingiva, tongue, buccal mucosa, hard palate, and floor of the mouth. OSCC incidence increases with age and is marked in older individuals and men. Globally, around 300,000 OSCC cases occur each year [3, 4]. Surgical management with adjunctive chemoradiotherapy is the mainstay of OSCC treatment. Although treatment of oral cancer has advanced over the last few decades, survival rates remain low, with 5-year survival rates of less than 50%, particularly among those with locally advanced disease [5, 6]. Carcinogenesis in OSCC is attributed to physical, chemical, or pathological aberrant stimulation of mucosal epithelial cells. Multiple biological processes and factors are implicated in OSCC carcinogenesis, and varied risk factors include genetic, environmental, and dietary factors. However, the exact molecular pathogenesis is not yet fully understood, and the search for biomarkers based on greater insight into biological mechanisms underpinning OSCC is an important research direction for the prevention, treatment, and prognosis prediction of OSCC.

Cancer cell invasion of basement membranes (BMs) have emerged as an essential area in OSCC research. BMs comprise a lamellar extracellular matrix with varying biological functions at different tissue sites [7]. BMs are complex in composition, which imposes challenges in studying their function and role. BMs are involved in resisting mechanical forces, maintaining the shape of tissues, and forming diffusion barriers. They also influence cell polarity, differentiation, migration, and survival. Increased expression of matrix metalloproteinase (MMP) genes may increase the risk of carcinogenesis. MMPs are often used as potential biomarkers for various cancers, including OSCC [8].

Recent advancements in high-throughput sequencing technologies and gene chip expression analyses have facilitated the deep and extensive capture of gene expression data. While bioinformatics studies have delineated mRNA expression profiles and identified many differentially expressed genes in OSCC, the relationship between basement membrane (BM)-associated gene expression levels and OSCC pathogenesis remains undefined.

In this study, we employed bioinformatic tools to investigate the potential roles of core BM-associated genes in OSCC. Our goal is to lay a theoretical foundation and offer data support for in-depth investigation into the molecular mechanisms underlying oral cancer.

## Methods

### Data collection

TCGA pan-cancer data were obtained from the UCSC Xena Gene expression and clinical metadata from the GTEx database (https://xenabrowser.net/datapages/). Tumor tissue data was obtained from the TCGA database (https://portal.gdc.cancer.gov/) and combined with data from paracancerous tissue using the TCGA and GTEx databases. Microarray datasets about OSCC were obtained from the GEO database (https://www.ncbi.nlm.nih.gov/geo/) and searched using the keyword “oral cancer”. The GSE42743 GEO [9] dataset and OSCC dataset GSE41613 based on the Affymetrix U133 plus 2.0 microarray was selected as a validation set for our study. This study included a control group comprising 3 paracancerous samples and a tumor group comprising 55 samples.

Using the ID of each sample, it was attempted to combine the expression levels of differentially expressed BMGs of each sample with the relevant prognostic outcomes. Prognosis-related genes were identified using univariate Cox regression (uni-Cox-reg) on differentially expressed BMGs from the TCGA (training dataset) and GEO (test dataset). The “maftools” R package was applied for the analysis of the mutation and the associated genes in the training dataset of OSCC samples. The analysis of prognosis-related genes was performed via the “glmnet” R package with the assistance of the least absolute shrinkage and selection operator (LASSO), which develop a prognosis-risk score model that could accurately predict overall survival rate (OS) of OSCC samples. The estimation of the penalty parameter of the model was carried out via the 10-fold cross-validation. The following formula was utilized for calculating the risk score of each sample: ${\rm{risk}}\,{\rm{score}} = \sum _{{\rm{i}} = 1}^{\rm{n}}{\rm{coef(i)}} \times {\rm{expre(i)}}$, in which “expre” represents the gene expression levels from the prognosis-risk score model, and “coef” indicates non-zero regression coefficients derived by LASSO regression analysis. Division of samples into high-risk score group and low-risk score group was carried out using the median value of risk scores. The log-rank test and Kaplan-Meier analysis were utilized to compare OS-related differences between the two above-mentioned groups. The “survivalROC” R package was utilized to plot the time-dependent ROC curves, which assisted in investigating the accuracy of predictability of the prognosis-risk score model. The validity and accuracy of the prognosis-risk score model were verified via the test dataset.

### Differential gene screening

Human HG38 gene annotation data were obtained, and a gene expression matrix was formed. Differentially expressed genes (DEGs) were screened using the R package (3.6.1) “Limma” package, with the criteria of |log2FC|>1 and *P* < 0.05 [10]. One-way Cox regression analysis was applied, and genes with prognostic value were subjected to the least absolute shrinkage and selection operator (LASSO)-Cox regression analysis to construct a risk-prognostic model.

### Construction of a basement membrane gene-based risk assessment model

Based on the differential basement membrane-associated genes (BMGs) obtained, LASSO-Cox regression was performed using the glmnet package in R to construct a risk model. BMG-based risk scores were assigned to each OSCC patient in the TCGA database. Validation was performed using microarray data from the GSE42743 [9] dataset, which included matched cancer and paracancerous tissue gene expression data and survival times for 24 OSCC patients. The correlation between the risk scores and clinicopathological features was analyzed for the TCGA cohort. Patients were categorized into low-risk and high-risk groups based on a cut-off set as the median risk score, and Kaplan-Meier survival curve analysis was done. In defining high-risk and low-risk groups within both the training and validation sets, we adhered to the same criteria, specifically the total survival time following initial diagnosis (measured in months).

### Receiver operating characteristics (ROC) and column plot

The sensitivity and specificity of the BMG-based risk model in predicting patient survival at years 1, 2, and 3 were assessed by plotting Receiver Operating Characteristics (ROC) curves using the R package ‘survivor ROC’. PCA ordination was performed to analyze the clustering of low-risk and high-risk samples. Columnar maps were constructed using clinical characteristics and risk models to predict survival at years 1, 2, and 3, and the accuracy of the columnar maps was assessed by C-index and calibration curves.

### Enrichment analysis of differentially expressed basement membrane-associated genes

Gene ontology (GO) and KEGG pathway functional enrichment analysis were performed to identify molecular and signaling pathways enriched by the differentially expressed BMGs. The cut-offs were set at *P* < 0.05, and the number of enriched genes ≥ 1. The analyses were performed using the “cluster profiler” R package.

### Univariate and multivariate Cox regression analysis

Prognostic values of basement membrane scores combined with clinicopathological features in the TCGA cohort were determined. Hazard Ratio (HR) values were calculated using Cox proportional regression. *P* < 0.05 indicated a statistically significant difference.

### Tumor immune landscape analysis

As immune infiltration is central to OSCC carcinogenesis, the tumor immune landscape and its correlation with the BMG-based risk scores were analyzed. CIBERSORT analysis was performed to quantify the distribution of immune cell infiltration based on gene expression data. Differences in the CIBERSORT scores of the high and low BMG-based risk score groupings were analyzed. Tumor Immune Dysfunction and Exclusion (TIDE) [11] score analysis was applied to understand the differences in cancer immunotherapy response based on BMG-based risk scores. The tumor microenvironment and proportion of tumor-infiltrating immune cells in the OSCC samples were sought using multiple approaches. The GSVA software package (Version 1.42.0) and the ‘MCP-Counter’ packages [12] that use the Single sample gene set enrichment analysis (ssGSEA) approach were applied. In addition, the ESTIMATE [13] and TIMER [14] algorithms were also applied to quantify immune and stromal cell infiltrates. The correlation between tumor-infiltrating immune cells and BMG-based risk scores was evaluated using Spearman correlation coefficient analysis.

### Single-gene disease-free survival (DFS) analysis

The 69 oral cancer samples from the TCGA database were used for single-gene DFS analysis. Samples were classified into high or low DEG expression groups according to FPKM > 1 or < 1 based on the expression level of each gene in each sample. Genes with p-values < 0.05 were considered as DEGs. Survival curves for the DEGs were plotted using the R package survival with the Kaplan-Meier method, and differences were tested using the log-rank method.

### Quantitative real-time PCR (qRT-PCR) experimental validation

Based on the results, a set of 3 BMGs LAMA3, MMP14, and GPC2 were considered for experimental validation. As laminin is a crucial basement membrane protein for membrane formation and maintenance, cell migration, and mechanical signal transduction, we focused on laminin for experimental validation. OSCC and patients’ paracancerous tissues from clinical samples (*n* = 10) were used for validation analysis with qRT-PCR, and the GAPDH was used as an internal reference gene to standardize the expression level of LAMA3. In brief, total RNA was extracted using the trizol method and assayed for RNA concentration and purity using a nanodrop 2000. The RNA was reverse transcribed to cDNA using a reverse transcription kit (item no. K1622, Thermo, USA) and then amplified using a Q-PCR kit (item no. 12,574,026, Thermo, USA) on a BioRad CFX384 real-time PCR machine. Analysis was performed using the 2 ^(−**△△**CT)^ method.

The primer sequences used were as follows:

LAMA3_F: GATTGA ATTGAGCACCAGCGATAGC;

LAMA3_R: GATGAGAAGCCGTAGTCCAGAGTTG;

GAPDH_F: AACAGCGACACCCACTCCTC;

GAPDH_R: CATACCAGGAAATGAGCTTGACAA.

### Prediction of IC50 value

The pRRophetic R package was employed to predict the IC50 (half maximal inhibitory concentration) of common chemotherapeutic drugs(PMID: 25,229,481). IC50 indicates a substance’s efficacy in terms of inhibiting particular biochemical or biological functions. We employed Wilcoxon signed-rank test to assess group differences. Using the “pRRophetic”, “limma”, “ggpub”, and “ggplot2” R packages, compounds that could be used for OSCC treatment were predicted.

## Results

### Differentially expressed basement membrane-associated genes in tumor and paracancerous tissues of oral cancer patients

**Fig. 1:**
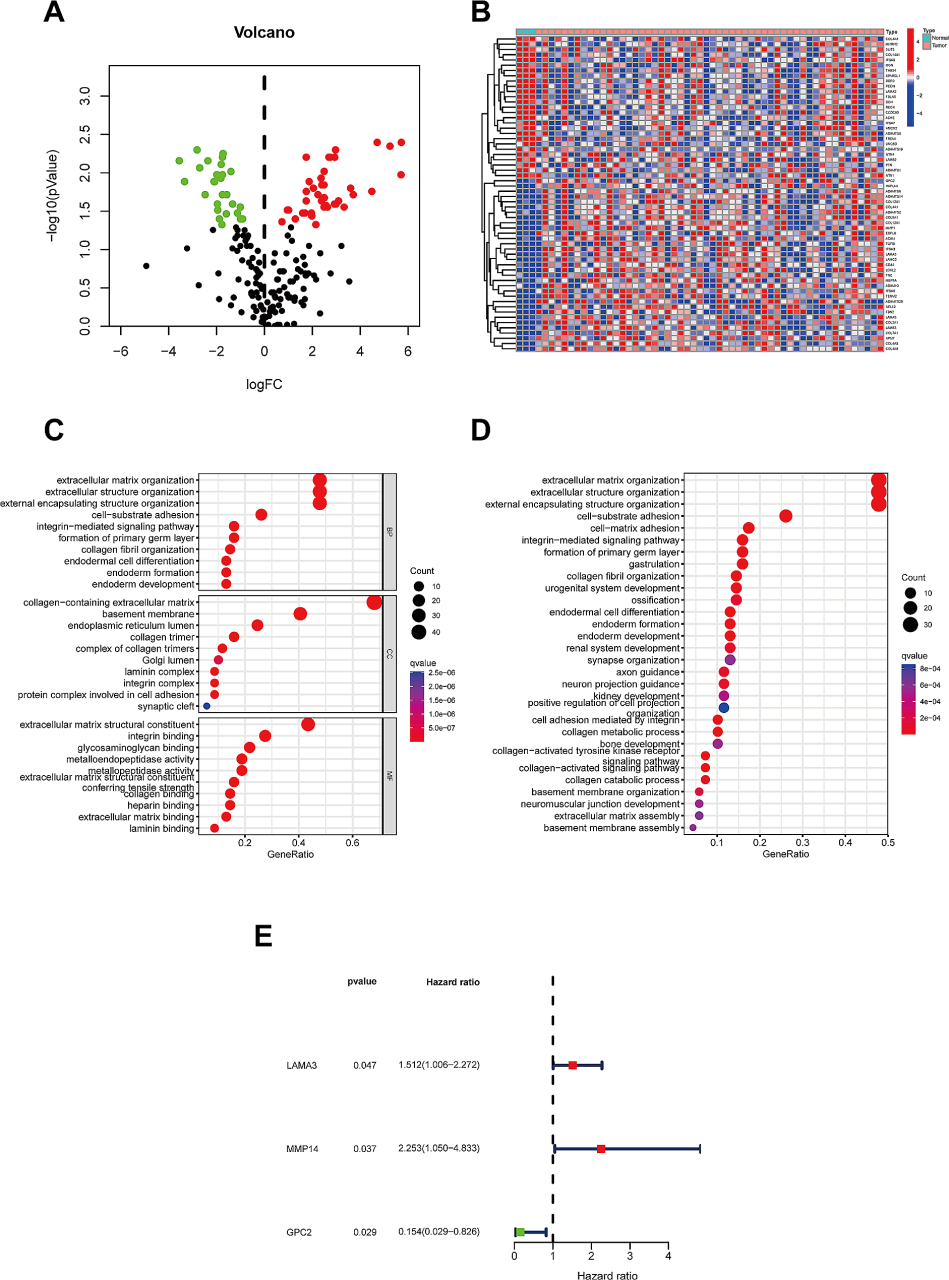
Differentially expressed BMGs in oral cancer cases. **A**. The volcano plot of 69 BMGs with significant differential expression. **B**. Heatmap depicting the expression levels of 69 BMGs in paracancerous and tumor tissues. **C-D**. Enriched GO terms and KEGG pathways in 69 BMGs. **E.** Forest plot depicting univariate-Cox-reg analysis of the association of 3 BMGs with survival prognosis

A total of 69 differentially expressed genes associated with basement membranes (BMGs) were obtained (*P* < 0.05, |log2FC)|>1.0). 42 genes were expressed at significantly higher levels, and 27 genes were expressed at lower levels than those in paracancerous tissues (Fig. 1A and B), which indicated an association of OSCC occurrence with BMGs. GO functional annotation analysis showed that the main enriched biological processes among the differential BMGs included extracellular matrix organization, extracellular structure organization, external encapsulating structure organization, cell-substrate adhesion, integrin-mediated signaling pathway, formation of primary germ layer, collagen fibril organization, endodermal cell differentiation, endoderm formation, endoderm development. The main cellular component-related enriched functions included collagen-containing extracellular matrix, basement membrane, endoplasmic reticulum lumen, collagen trimer, complex of collagen trimers, Golgi lumen, laminin complex, integrin complex, protein complex involved in cell adhesion, and synaptic cleft. The major enriched molecular function included extracellular matrix structural constituent, integrin binding, glycosaminoglycan binding, metalloendopeptidase activity, metallopeptidase activity, extracellular matrix structural constituent, conferring tensile strength, collagen binding, heparin-binding, extracellular matrix binding, laminin-binding (Fig. 1C).

KEGG pathway analysis showed that the enriched signaling pathways mainly comprised extracellular matrix organization, extracellular structure organization, external encapsulating structure organization, cell-substrate adhesion, and integrin-mediated signaling pathway (Fig. 1D). We conducted risk modeling using genes that were differentially expressed in both the TCGA and GEO datasets, and identified these three genes as key components of the risk models. Our decision was informed by their significance in initial differential expression analyses, their documented biological functions in the literature, and their established roles in oral cancer and other cancer types. Previous studies have linked the LAMA3 gene to cancer aggressiveness, metastatic potential, and patient survival, while bioinformatics prescreening highlighted its unique expression patterns and prognostic relevance. Whatmore, one-way Cox regression analysis showed that three BMGs, LAMA3, MMP14, and GPC2, were significant prognostic predictors in oral cancer patients (Fig. 1E).

### Construction of a BMGs prognostic risk score model

**Fig. 2:**
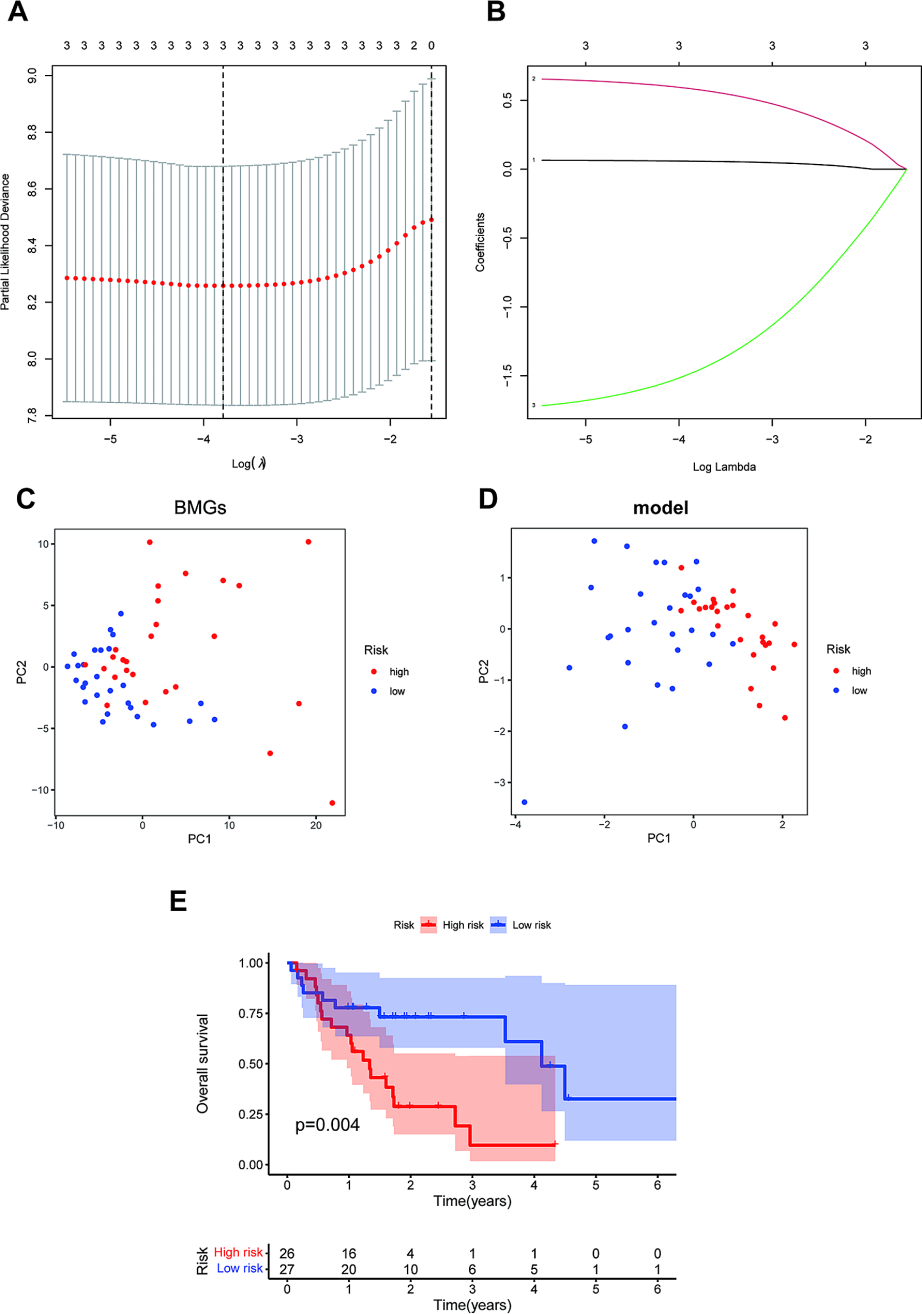
Developing a PRS model using 3 BMGs. **A**. Identification of 3 BMGs for a prognostic risk score (PRS) model. **B**. The LASSO coefficients of the 3 BMGs. **C-D**. PCA plot according to low- and high-risk scores. **E**. Kaplan–Meier survival curves were plotted for overall survival

A prognostic risk score (PRS) model centered on BMG expression was developed through LASSO-Cox regression analysis, utilizing differentially expressed genes with noteworthy prognostic values (Fig. 2A and B). We developed risk models using differentially expressed genes shared between the TCGA and GEO datasets, discovering that only three BMG genes(LAMA3, MMP14, and GPC2)were instrumental in constructing the models. Their coefficients were 0.057959721, 0.577569041 and − 1.459461854, respectively. PCA unveiled distinct clustering of BMGs per high- and low-risk groups, suggesting that numerous aberrantly expressed BMGs play a role in the evolution of oral cancer (Fig. 2C and D).

Risk scores were assigned to each sample within the TCGA-STAD cohort, categorizing patients into high-risk (*n* = 26) and low-risk (*n* = 27) groups — a division established based on the median risk score. Kaplan-Meier analysis further affirmed the model’s validity, as it illustrated a markedly reduced survival span in the high-risk group (Fig. 2E). This result underscores the prognostic utility of the BMG risk model in foretelling the survival outcomes for individuals with oral cancer.

### Prognostic value of basement membrane score combined with clinicopathological features in the TCGA cohort

**Fig. 3:**
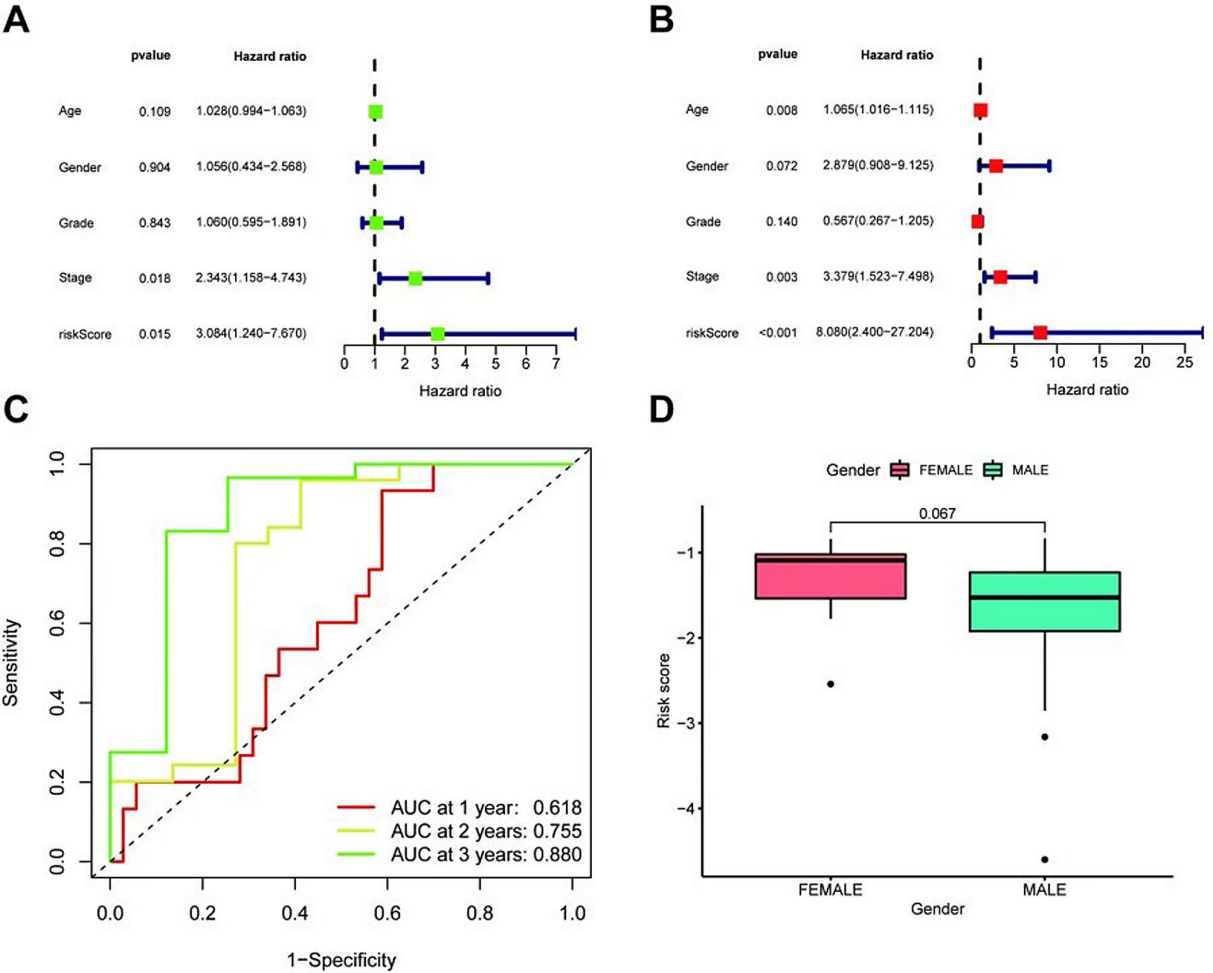
The prognostic value of the BMG-based risk score combined with clinicopathological features in the TCGA cohort. **A-B**. The multivariate-Cox and univariate-Cox regression analyses for risk score and clinical variables associated with overall survival. **C**. The 1-, 2-, and 3-year ROC curves depict the association of the risk scores with clinical characteristics. **D**. Boxplots depicting risk scores by gender

BMG-based risk scores were subjected to Cox regression to examine whether they could be independent prognostic risk factors. Univariate analysis showed that tumor stage and risk score were factors associated with survival prognosis (Fig. 3A). The multifactorial analysis showed that age, tumor stage, and risk score were independent prognostic risk factors (Fig. 3B). The ROC curve analysis showed that the area under the curve (AUC) was 0.618 for one-year survival, 0.755 for two years, and 0.880 for three years (Fig. 3C), indicating good prognostic performance. In the TCGA cohort, the High-Risk group exhibited a poorer prognosis(which was defined in this study as both death and neck metastasis) compared to the Low-Risk group. The risk scores did not differ significantly between males and females (*P* = 0.067, Fig. 3D), although higher risk scores were observed for females.

### Immunological subtypes and TIDE scores

**Fig. 4:**
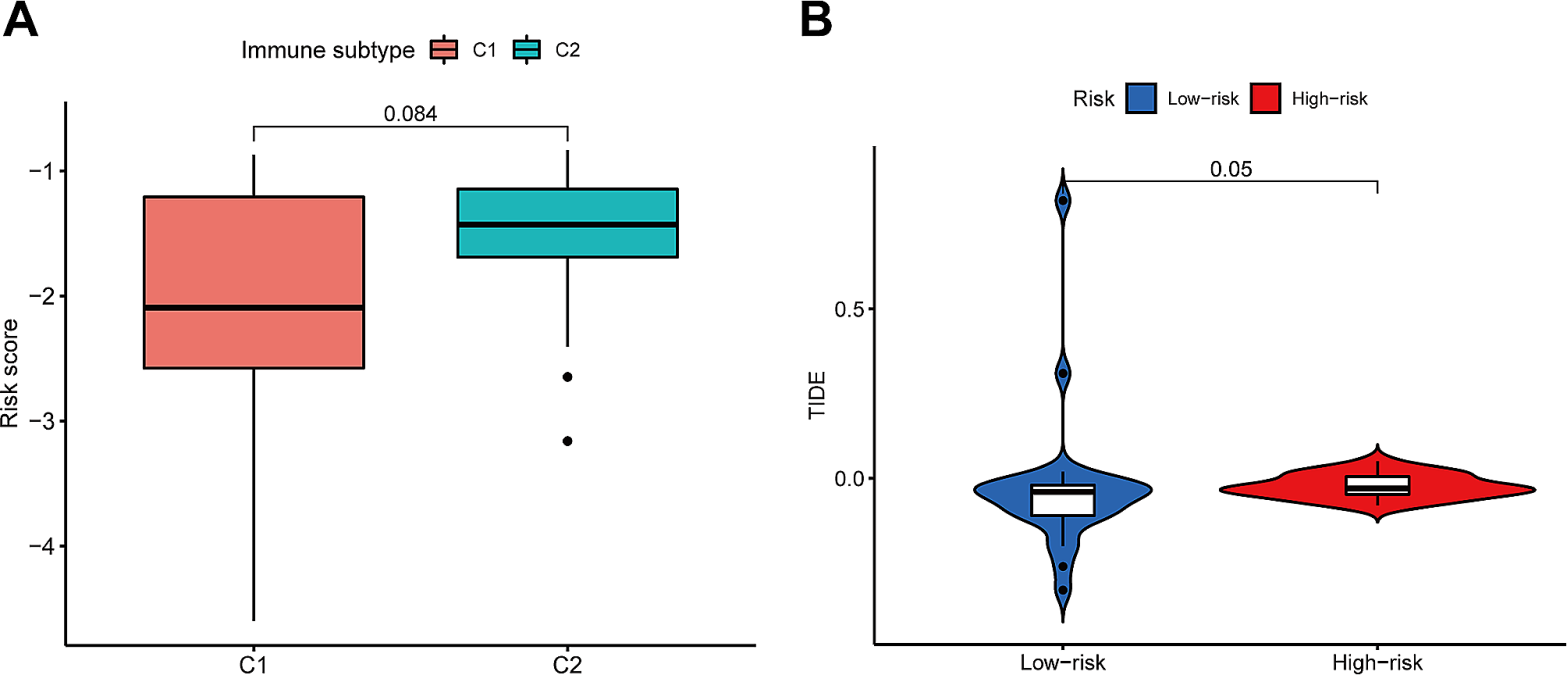
Immune subtype and TIDE scores. **A**. Boxplots depicting risk score by immune subtype. **B**. The comparison of TIDE scores between low- and high-risk groups

No statistically significant difference between the C1(Wound Healing) and C2 (IFN-gamma Dominant)immune subtype groups was seen for risk scores, *P* = 0.084 (Fig. 4A). TIDE analysis was applied to analyze the potential differences in immunotherapy outcomes between molecular subtypes, and a significant difference in scores between the high- and low-risk groups was noted (Fig. 4B).

### Basement membrane genes and relevance to immunotherapy

**Fig. 5:**
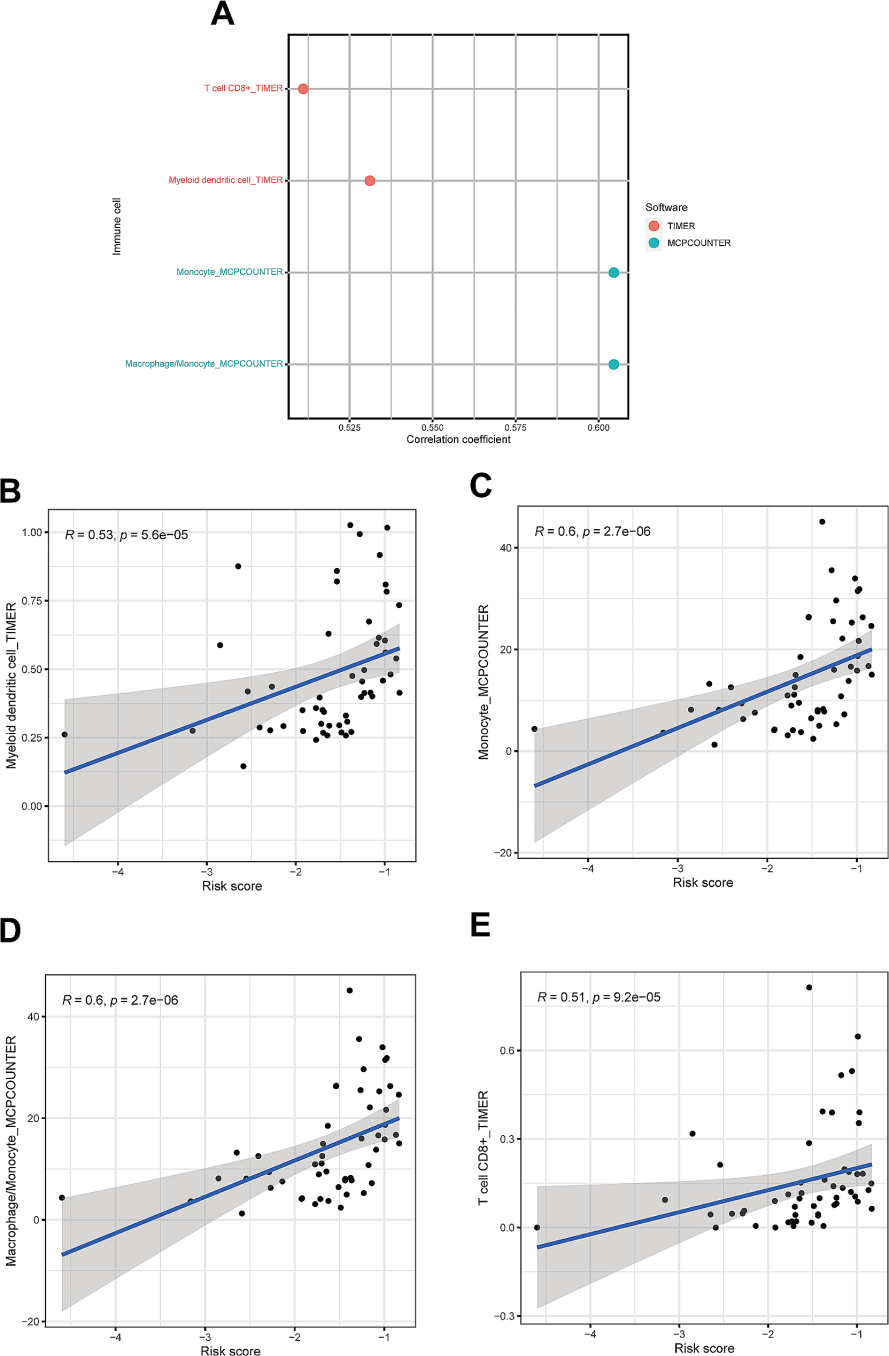
Tumor immune landscape and BMG-based risk scores. **A**. Bubble plots depicting the correlation coefficients for infiltrating immune cells in BMG-based risk groups. **B-E**. Scatter plots depicting the correlation analysis of risk scores with various infiltrating immune cells

Bubble plots were drawn to depict the association of the immune cells and risk scores (Fig. 5A). Bone marrow cells, T cells, monocytes, and macrophages were positively correlated with risk scores using TIMER and MCPCOUNTER analyses. Monocytes and macrophages/monocytes are quantified using MCPCOUNTER, while CD8^+^ T cells and myeloid dendritic cells are quantified using TIMER with specific correlation coefficients provided in the Table 1. Several immune cells, including bone marrow cells, macrophage/monocyte, and T cells, were correlated with risk scores (Fig. 5B-E).

**Table 1:** Correlation coefficients

immune	cor	pvalue
T cell CD8^+^_TIMER	0.511056907	9.22E-05
Myeloid dendritic cell_TIMER	0.531124012	5.59E-05
Monocyte_MCPCOUNTER	0.604579906	2.75E-06
Macrophage/Monocyte_MCPCOUNTER	0.604579906	2.75E-06

### ssGSEA for immune cell and immune function scores

**Fig. 6:**
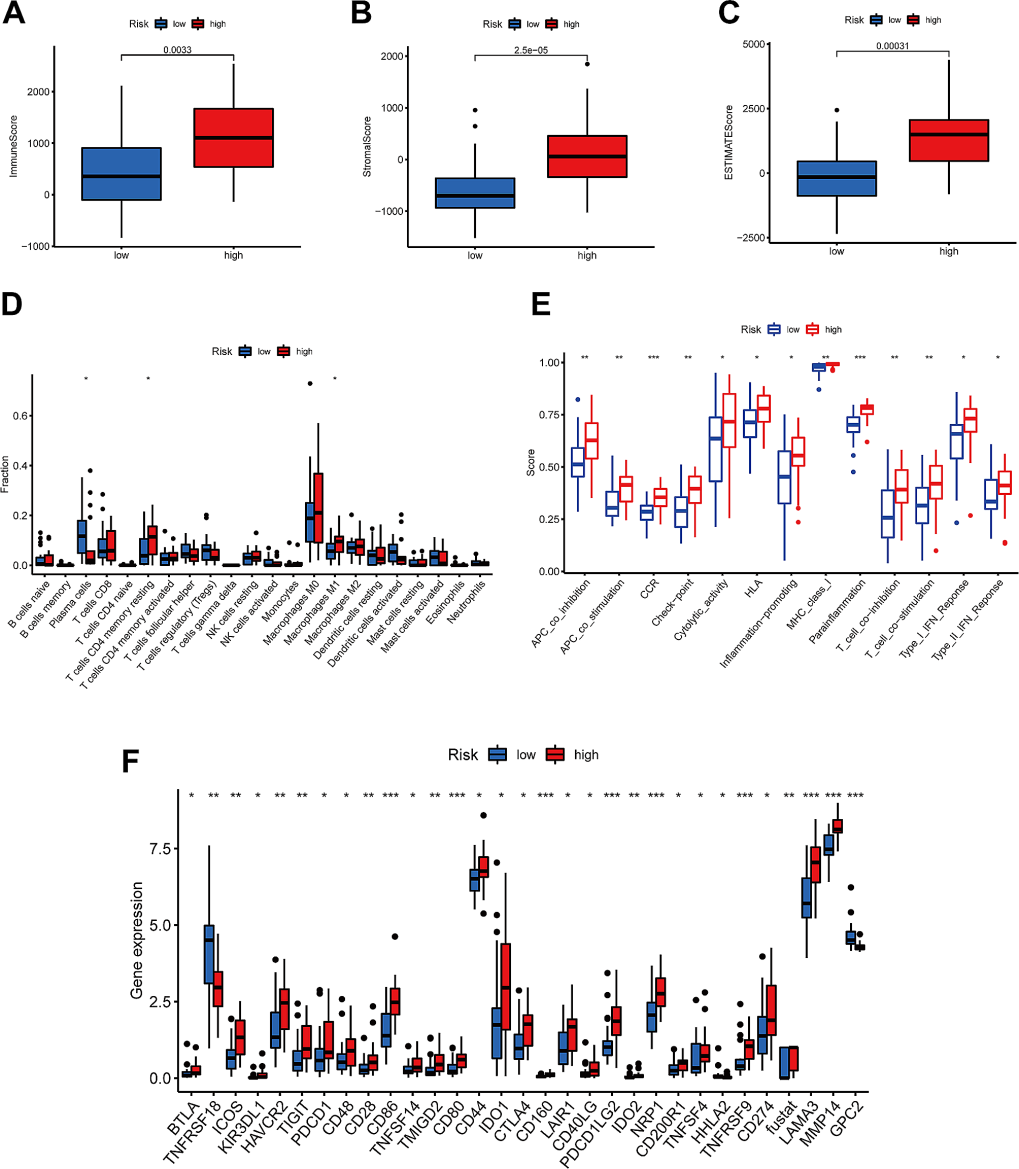
Predicted tumor immunotherapy-related molecules associated with BMG-based risk scores. **A-C**. The comparison of ESTIMATE immune scores between low- and high-risk groups. **D-E**. The relative proportion of infiltrating immune cells and functions was assessed using ssGSEA. **F**. The expression levels of 18 immune checkpoints in the two risk groups. * *P* < 0.05, ** *P* < 0.01, and *** *P* < 0.001

“ESTIMATE” was applied to analyze the tumor microenvironment and the ratio of stromal-immune cells. Higher immune, stromal, and overall ESTIMATE scores in the high-risk group of OSCC patients indicated a greater immunological activation (Fig. 6A-C). Higher enrichment scores for various immune cells (B cells, macrophages, T cells CD8, NK cells, etc.) were also noted in the high-risk group (Fig. 6D). Immune checkpoint analysis showed that several immune checkpoint-related functions, including APC co-stimulation, CCR, checkpoints, MHC class 1, HLA, para-inflammation, T cell co-stimulation, T cell co-inhibition, type I IFN response and type II IFN response were also significantly higher in the high-risk group (Fig. 6E). These results suggest that BMGs are also closely implicated in immune cell regulation. In addition, the proportion and function of infiltrating immune cells significantly differed between risk score groups (Fig. 6F).

### Immunotherapeutic drug sensitivity analysis

**Fig. 7:**
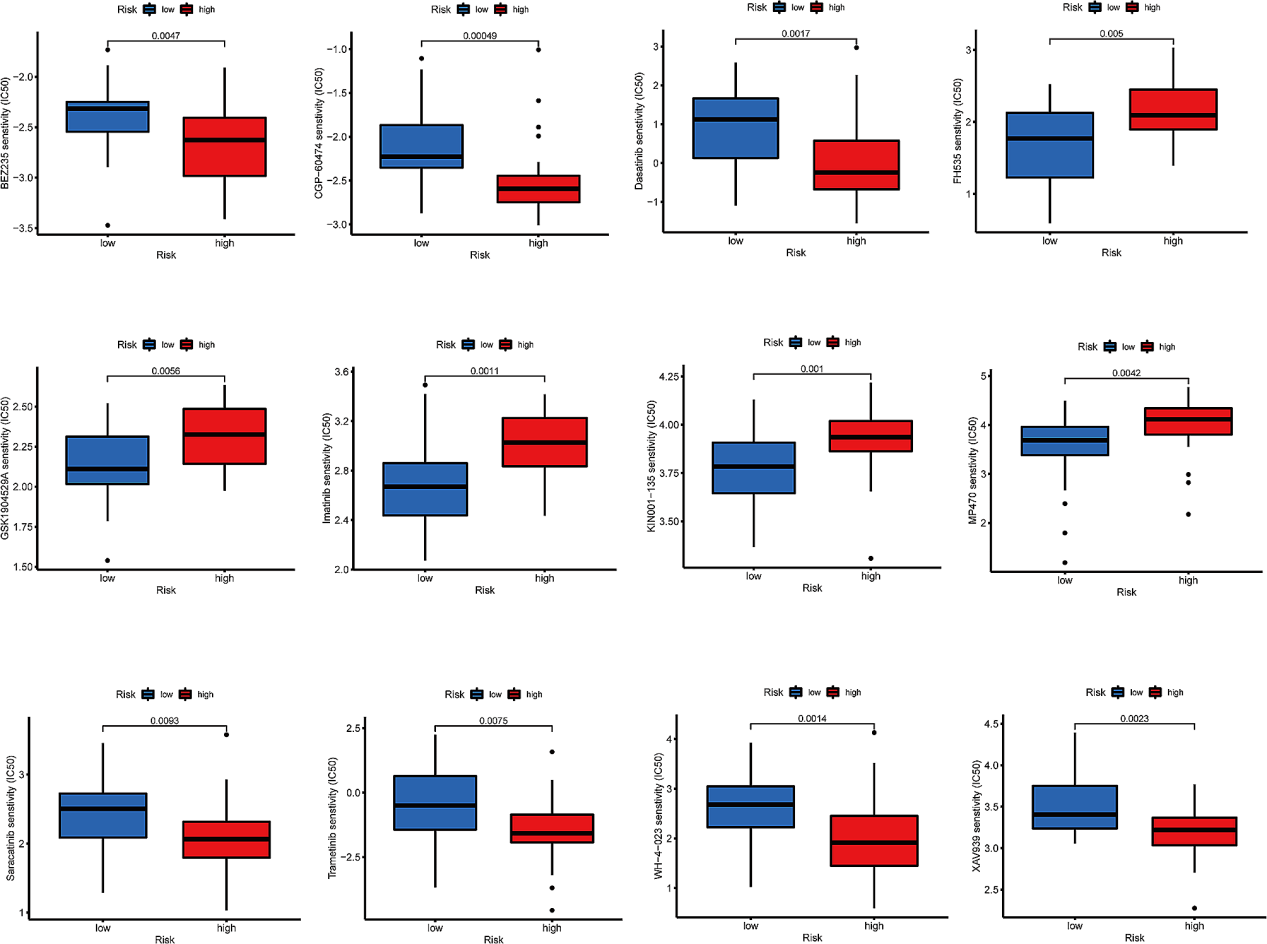
Immunotherapy analysis for risk groups. The IC50 values of 12 immunotherapeutic, chemical, or targeted medicines used in risk groups were tested

In the analysis of exploring differences in sensitivity to immunotherapeutic drugs between the two identified risk groups, our results revealed that the compounds BEZ235, CGP-60,474, Dasatinib, Saracatinib, Trametinib, WH-4-023, and XAV939 demonstrated a significant variation in predicted efficacy. Specifically, these drugs showed enhanced sensitivity in the high-risk group compared to the low-risk group. This suggests a potential pharmacological vulnerability in the high-risk group that could be exploited for therapeutic advantage (Fig. 7). Although several of these drugs are currently at the research stage, these findings imply that BMG expression patterns may influence precision immunotherapy. This study identifies novel therapeutic targets for oral cancer in the absence of empirical evidence and introduces innovative concepts for the development of new drug targets in future clinical treatments.

### Prognostic value of LAMA3 in OSCC

**Fig. 8:**
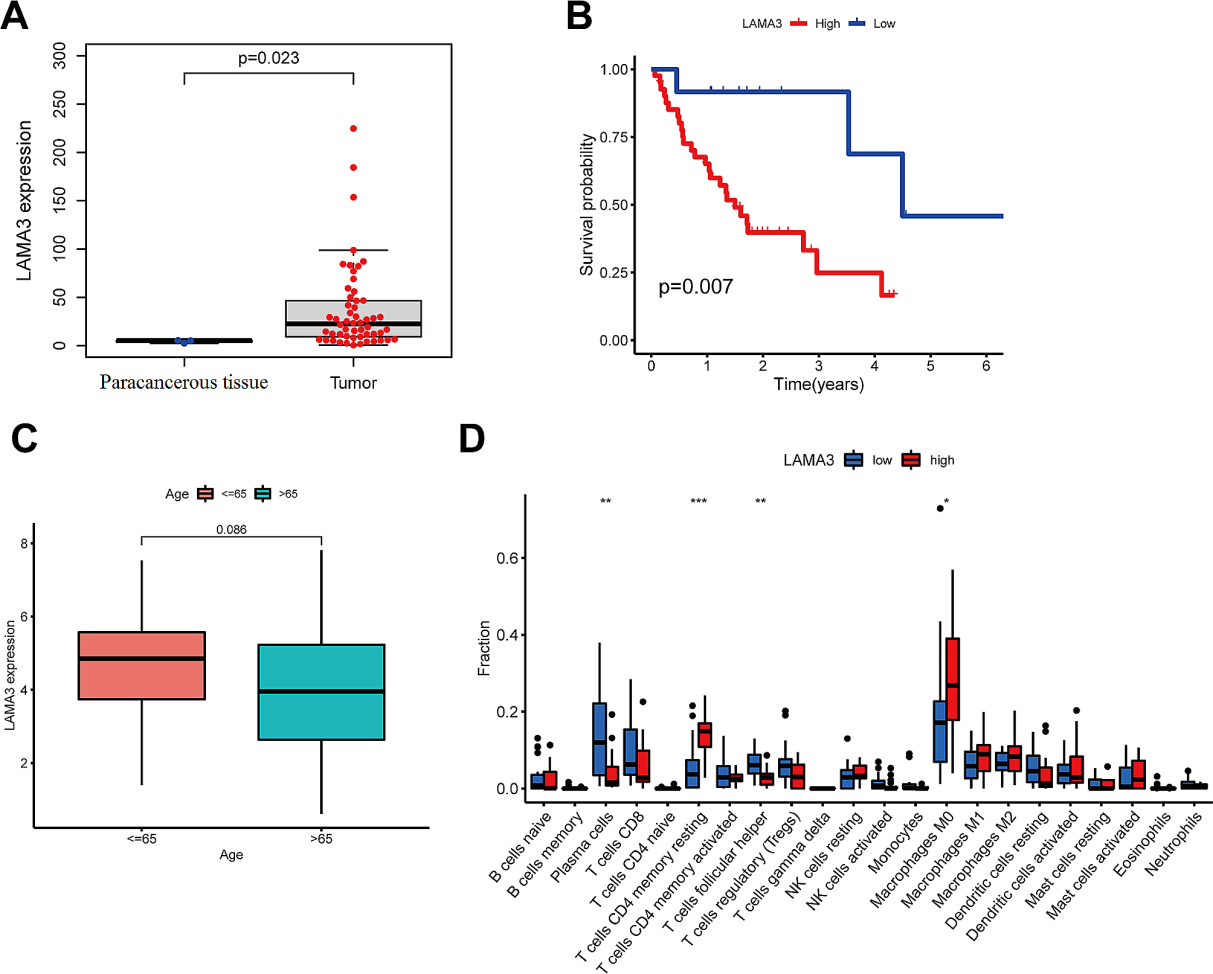
The prognostic BMGs were analyzed by univariate-Cox-regression analysis. **A**. Boxplots summarizing differences in LAMA3 expression in cancerous and paracancerous specimens from the same patients (*P* = 0.023, by the WRS test). **B**. Survival analysis conducted using LAMA3 expression levels as a predictor. Patients were grouped into high- or low-expression-level groups based on a cut-off set as the median expression level (*P* < 0.001, the log-rank test). **C**. Boxplots showing differences in LAMA3 expression level by age group. **D**. The relative proportion of infiltrating immune cells determined using ssGSEA in patients with high and low LAMA3 expression. * *P* < 0.05, ** *P* < 0.01, and *** *P* < 0.001

LAMA3 expression was significantly higher in cancerous tissues of OSCC patients than that in paracancerous tissues (*P* = 0.023) (Fig. 8A). Patients were stratified into high and low expression groups based on the median LAMA expression as the cutoff value. Kaplan-Meier survival analysis for the high-risk and low-risk groups showed that LAMA3 expression predicted significantly longer overall survival in the low-expression group than that in the high-expression group (*P* = 0.007) (Fig. 8B). One-way Cox regression analysis with age and LAMA3 expression as predictors showed that LAMA3 expression levels were independent of age (*P* = 0.086) (Fig. 8C). The proportion of different immune cells with LAMA expression levels in the high- and low-risk groups were analyzed by ssGSEA, showing higher enrichment of various immune cells (B cells naive, T cells CD4 memory resting, macrophages, NK cells, etc.) in the high-risk group (Fig. 8D). These findings implied that increased LAMA3 expression may be an independent poor prognostic factor for oral cancer.

### LAMA3 expression in OSCC cancer and paracancerous tissue

**Fig. 9:**
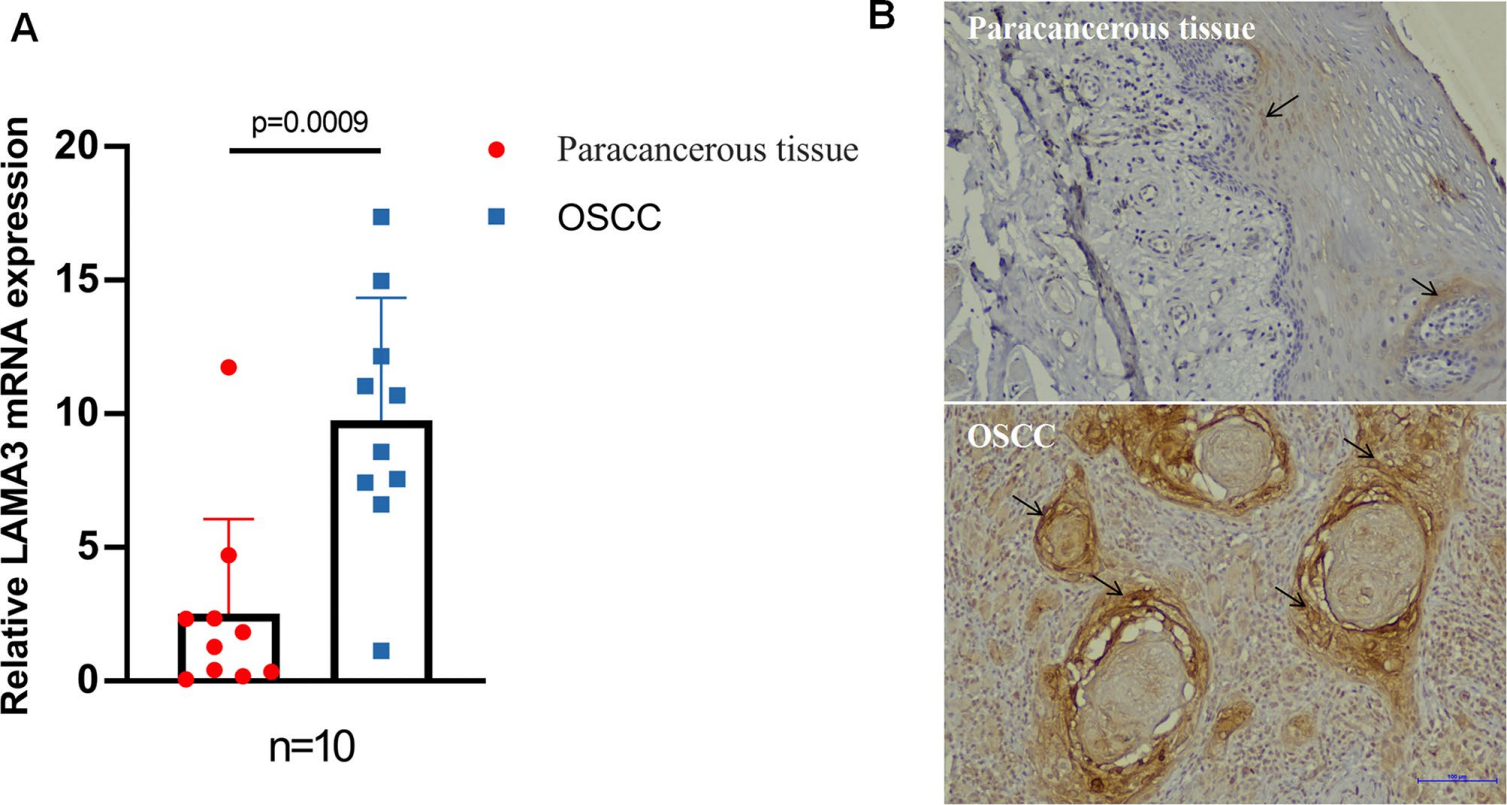
LAMA3 expression in OSCC cancer and paracancerous tissues. **A**. qRT-PCR was used to quantify LAMA3 gene expression in cancerous and paracancerous tissues. The expression of the LAMA3 gene in cancerous tissue was significantly higher than that in paracancerous tissues. (*n* = 10, *P* = 0.0009). **B**. Immunohistochemical staining was used to detect LAMA3 protein expression in cancerous and paracancerous tissue. Arrow showing LAMA3 positivity, scal bar = 100 μm

OSCC and paracancerous tissues from patients (*n* = 10) were randomly selected for qRT-PCR, and the level of LAMA3 mRNA in oral cancer tissues was significantly higher than that in paracancerous tissues (*P* < 0.05) (Fig. 9A). Immunohistochemical staining results were concordant, showing significantly higher expression of LAMA3 protein in cancerous tissue than that in paracancerous tissue (Fig. 9B).

## Discussion

Cancer cell proliferation is a foundational element in cancer metastasis, the primary contributor to unfavorable survival outcomes in cancer patients [15]. The basement membrane is integral in facilitating tumor cell shedding, a process crucial for metastasis where cells become less adherent and more invasive, ultimately being released into the cytoplasmic stroma and potentially establishing new tumors in distant organs [16, 17]. Given the high metastatic rate of oral cancer, resulting in often dismal postoperative prognoses, exploring the role of genes associated with the basement membrane in clinical outcomes is imperative to uncover new avenues for clinical and therapeutic interventions.

Despite the established involvement of a protease-dependent pathway mediated by matrix metalloproteinases (MMPs) in basement membrane functionalities, MMP inhibitors have shown limited effectiveness in reducing mortality rates among cancer patients [18]. This highlights an urgent need to thoroughly examine genes and proteins related to the basement membrane to identify new therapeutic targets.

We successfully constructed an oral cancer risk model based on basement membrane-associated genes by leveraging publicly available data from the TCGA database using Cox regression and Lasso regression analysis. The risk scores were validated using an independent dataset from the GSE42743 cohort, and we showed that the high-risk group scores predicted worse prognostic survival. The AUC values suggested a good prognostic performance of the BMGs-based risk score for overall survival.

The immune microenvironment is an essential determinant of tumor biology. It is currently the focus of attention in tumor-related fields. We showed an enrichment of B cells, macrophages, neutrophils, T helper cells, and NK cells immune cells in the high-risk group. Macrophage and monocyte infiltration predict poor prognosis in oral cancer patients in the high-risk group. The loss of basement membrane proteins has been associated with increased cancer-related inflammatory cell infiltration. Further research is needed to uncover the crosstalk between basement membrane proteins [19] and macrophages in epithelial, stromal cell compartments in OSCC. Immune function scores in the high-risk group also revealed significantly higher APC co-stimulation, CCR, checkpoint, MHC class 1, HLA, paraneoplastic, T-cell co-stimulation, T-cell co-inhibition, type I IFN response, and type II IFN response, reflecting that molecular alterations in the basement membrane accompany aberrant immune activation in OSCC. Of note, CD4 T-cell fractions were enriched in the high-risk group, and an increased CD4/CD8 T-cell ratio is linked to OSCC invasion and associated with a pro-inflammatory milieu [20]. The immune checkpoint analysis and drug sensitivity indicated several checkpoint genes were expressed at higher levels in the high-risk group, reflecting regulatory roles of BMGs in immune checkpoint regulation and may have relevance to precision medicine, thus warrants further investigations. Mainwhile, Our research will contribute to exploring the effects of immunological markers on drug sensitivity, and the potential effects of using different drugs in combination. It will aid in discovering more effective combination treatment strategies, which is of great significance for guiding current and future treatment methods.

Enrichment analysis demonstrated that the risk BMGs were mainly enriched in extracellular matrix-containing tissue, extracellular structural tissue, external encapsulating structure organization, cell-substrate adhesion, integrin-mediated signaling pathway, basement membrane, etc. KEGG pathway analysis showed activation of the extracellular matrix, extracellular structural tissue, cell-substrate adhesion, and integrin-mediated signaling pathways, all involved in the shedding, invasion, and migration of oral cancer cells and are essential components of the extracellular matrix. BMGs may contribute to the development of oral cancer by altering the extracellular matrix, thus inducing tumor microenvironment changes.

We demonstrated and verified the prognostic significance of the BMG, LAMA3 (Laminin subunit alpha 3), encoding for Laminin-332 (LM-332), a significant member of the laminin family. LAMA3 has variable gene expression patterns in different cancer cells, with high expression levels in hepatocellular carcinoma and pancreatic cancer and lower expression levels in gastric, breast, and prostate cancer. However, there are few reports on LAMA3 in oral cancer [21–24]. We found that LAMA3 expression was significantly higher in cancerous tissues of OSCC patients compared to paracancerous tissues. Kaplan-Meier survival analysis showed that LAMA3 predicted worse overall survival in the high-expression group, verified by univariate and multifactorial Cox regression analyses. Our results are consistent with those of Tanis et al. [25], who performed genome-wide expression profiling of OSCC and reported LAMA3 as an early marker of OSCC of oral cancer. Our findings suggest that increased levels of LAMA3 expression may also be an independent predictor of poor prognosis and should be validated in longitudinal studies. In head.

and neck cancer (HNC), the invasion-associated molecules LAMA3, LAMC2, THBS1, IGF1R, PDGFB, and transforming growth factor β1 can serve as prognostic indicators or molecular therapeutic targets to improve the survival rates of HNC. These related molecular pathways may offer new strategies for further therapeutic applications [26]. The induction of LAMA3 in head and neck squamous cell carcinomas is influenced by hypoxia, and the splice variant LAMA3-A, whose differential expression is associated with tumor hypoxia, cannot be induced by hypoxia independently, but the exact pathways involved in the expression of this transcript are not yet clear [27]. These results help provide direction for our research.

We acknowledge that LAMA3 has at least two alternatively spliced mRNA forms, with one being implicated in metastasis while the other is not. Due to the nature of the data obtained from the TCGA database, our analysis did not differentiate between these isoforms. Consequently, our results reflect the collective expression of LAMA3 without distinguishing its splice variants. Future studies, equipped with isoform-specific expression data, are warranted to dissect the contributions of each LAMA3 splice variant in cancer progression and prognosis.

In our study, in order to be consistent with the sample size in validation set GSE42743, we selected three paracancerous control samples to determine the differentially expressed basement membrane genes that had been identified in patients with oral cancer. Although this choice enables us to compare our results effectively with independent datasets, we acknowledge that using a relatively small number of control samples may limit our judgement on the accuracy of differentially expressed genes. To alleviate this problem and improve the accuracy of the analysis, we used the weighted observations method in the limma package. By assigning a higher weight to groups with smaller sample sizes, such as the group of paracancerous tissue samples, we were able to mitigate to some extent the possible impact of sample imbalance. We recognize that despite the use of such mitigation measures, sample size imbalances remain a concern in our study. Nevertheless, our study provides preliminary insights into the potential role of basement membrane genes in the development of oral cancer, laying the foundation for more extensive and in-depth research in the future.

Meanwhile, when conducting this study, we faced some other limitations. Firstly, due to the limitation in data availability, our study only analyzed 69 oral cancer samples from the TCGA database for single-gene disease-free survival (DFS) analysis. Although the TCGA database provided samples with mRNA expression data from more than 100 patients who were followed up for overall survival for at least 1000 days after diagnosis, only some samples were included in the analysis because this study focused on samples with complete DFS information. We admit that the limited sample size may have a certain impact on the results and generalization of the study. Secondly, regarding the selection of division criteria for gene expression levels, this study adopted cut-off criteria of FPKM > 1 or < 1 to define the expression groups of high and low differentially expressed genes. This simple cut-off criterion has its advantages, but it may also limit the ways in which we can identify more subtle associations with disease prognosis. We did not establish a fold change threshold for differentially expressed genes, a decision that, although designed to simplify the initial exploration process, may not fully reveal the complexity of gene expression changes. We plan to overcome these limitations in future studies by expanding the sample size and applying more stringent criteria for identification of differentially expressed genes. Increasing the sample size not only improves the statistical power of the study, but also helps us to understand the association between gene expression and disease-free survival in oral cancer patients more accurately. At the same time, using more detailed gene expression analysis methods, such as setting specific fold change thresholds, will help us to discover more subtle and potentially biologically significant gene expression differences. In summary, although the current study methodology and design have certain limitations, we believe that these preliminary results can provide valuable insights and guidance for future research. We look forward to further exploring and validating these findings in future studies.

In addition, our study relied on publicly accessible databases and was lack of clinical or experimental validation for both the risk score and its constituent BMGs. It remains imperative to undertake experimental inquiries to elucidate the functional and molecular dynamics associated with the expression of these risk BMGs, facilitating a more comprehensive grasp of their significance in tumor biology. Consequently, the current results should serve as a foundation for generating further hypotheses and guiding subsequent experimental research.

## Conclusions

In conclusion, our research affirmed the efficacy of a basement membrane gene-based risk model in predicting survival outcomes in oral cancer. We unveiled various regulatory pathways and tumor microenvironment characteristics linked to heightened expression of basement membrane biomarker genes. Notably, a correlation was observed between the basement membrane risk gene expression and checkpoint gene expression patterns in oral cancer, suggesting a potential role in selecting immunotherapy agents. Moreover, we confirmed the high expression of LAMA3, a critical prognostic BMG, in clinical OSCC tissue samples. These bioinformatics-driven findings and the derived risk model call for additional validation through expansive clinical cohorts and longitudinal studies to further substantiate their potential in enhancing oral cancer prognosis and treatment strategies.

## Data Availability

The datasets generated and analyzed during the present study are available from the corresponding author on reasonable request.

## Abbreviations

BM: Basement membrane
BMs: Basement membranes
BMGs: BM-associated genes
DEGs: Differentially expressed genes
GO: Gene ontology
HR: Hazard Ratio
HNC: Head-neck cancers
LASSO: Least absolute shrinkage and selection operator
LM-332: Laminin-332
MMP: Matrix metalloproteinase
MMPs: Matrix metalloproteinases
OSCC: Oral squamous cell carcinoma
ROC: Receiver Operating Characteristics
TIDE: Tumor Immune Dysfunction and Exclusion
PRS: Prognostic risk score
qRT-PCR: quantitative real-time PCR
ssGSEA: Single sample gene set enrichment analysis

## References

[1] Yakop F, Abd Ghafar SA, Yong YK (2018). Silver nanoparticles clinacanthus nutans leaves extract induced apoptosis towards oral squamous cell carcinoma cell lines. Artif Cells Nanomed Biotechnol.

[2] Sung H, Ferlay J, Siegel RL (2021). Global cancer statistics 2020: Globocan estimates of incidence and mortality worldwide for 36 cancers in 185 countries. CA Cancer J Clin.

[3] Petersen PE (2003). The world oral health report 2003: continuous improvement of oral health in the 21st century–the approach of the who global oral health programme. Community Dent Oral Epidemiol.

[4] Wong T, Wiesenfeld D (2018). Oral cancer. Aust Dent J.

[5] Neville BW, Day TA (2002). Oral cancer and precancerous lesions. CA Cancer J Clin.

[6] Parkin DM, Bray F, Ferlay J (2005). Global cancer statistics, 2002. CA Cancer J Clin.

[7] Sekiguchi R, Yamada KM (2018). Basement membranes in development and disease. Curr Top Dev Biol.

[8] Scurry WC, Stack BC (2007). Role of metalloproteins in the clinical management of head and neck squamous cell carcinoma. Head Neck.

[9] Lohavanichbutr P, Méndez E, Holsinger FC (2013). A 13-gene signature prognostic of HPV-negative OSCC: discovery and external validation. Clin Cancer Res.

[10] Jayadev R, Morais M, Ellingford JM (2022). A basement membrane discovery pipeline uncovers network complexity, regulators, and human disease associations. Sci Adv.

[11] Jiang P, Gu S, Pan D (2018). Signatures of T cell dysfunction and exclusion predict cancer immunotherapy response. Nat Med.

[12] Becht E, Giraldo NA, Lacroix L (2016). Estimating the population abundance of tissue-infiltrating immune and stromal cell populations using gene expression. Genome Biol.

[13] Yoshihara K, Shahmoradgoli M, Martínez E (2013). Inferring tumor purity and stromal and immune cell admixture from expression data. Nat Commun.

[14] Li T, Fu J, Zeng Z (2020). TIMER2.0 for analysis of tumor-infiltrating immune cells. Nucleic Acids Res.

[15] Lee IS, Sahu D, Hur H (2021). Discovery and validation of an expression signature for recurrence prediction in high-risk diffuse-type gastric cancer. Gastric Cancer.

[16] Liu M, Yang J, Xu B et al. Tumor metastasis: Mechanistic insights and therapeutic interventions. *MedComm (2020)*. 2021;2:587–617. 10.1002/mco2.100 PMC8706758 34977870

[17] Banerjee S, Lo WC, Majumder P (2022). Multiple roles for basement membrane proteins in cancer progression and EMT. Eur J Cell Biol.

[18] Javadi S, Zhiani M, Mousavi MA (2020). Crosstalk between epidermal growth factor receptors (egfr) and integrins in resistance to egfr tyrosine kinase inhibitors (tkis) in solid tumors. Eur J Cell Biol.

[19] Caley MP, Martins VL, Moore K (2021). Loss of the laminin subunit alpha-3 induces cell invasion and macrophage infiltration in cutaneous squamous cell carcinoma. Br J Dermatol.

[20] Goertzen C, Mahdi H, Laliberte C (2018). Oral inflammation promotes oral squamous cell carcinoma invasion. Oncotarget.

[21] Ii M, Yamamoto H, Taniguchi H (2011). Co-expression of laminin β3 and γ2 chains and epigenetic inactivation of laminin α3 chain in gastric cancer. Int J Oncol.

[22] Kim BG, An HJ, Kang S (2011). Laminin-332-rich tumor microenvironment for tumor invasion in the interface zone of breast cancer. Am J Pathol.

[23] Gandellini P, Profumo V, Casamichele A (2012). miR-205 regulates basement membrane deposition in human prostate: implications for cancer development. Cell Death Differ.

[24] Sathyanarayana UG, Maruyama R, Padar A (2004). Molecular detection of noninvasive and invasive bladder tumor tissues and exfoliated cells by aberrant promoter methylation of laminin-5 encoding genes. Cancer Res.

[25] Tanis T, Cincin ZB, Gokcen-Rohlig B (2014). The role of components of the extracellular matrix and inflammation on oral squamous cell carcinoma metastasis. Arch Oral Biol.

[26] Chen YJ, Chang JT, You GR (2021). Panel biomarkers associated with cancer invasion and prognostic prediction for head-neck cancer. Biomark Med.

[27] Moller-Levet CS, Betts GN, Harris AL (2009). Exon array analysis of head and neck cancers identifies a hypoxia related splice variant of LAMA3 associated with a poor prognosis. PLoS Comput Biol.

